# The Psychometric Properties of the Meaning in Life Questionnaire (MLQ) in Patients with Life-Threatening Illnesses

**DOI:** 10.1155/2020/8361602

**Published:** 2020-01-28

**Authors:** Maede Naghiyaee, Bahman Bahmani, Ali Asgari

**Affiliations:** Department of Counseling, University of Social Welfare and Rehabilitation Sciences, Tehran, Iran

## Abstract

**Background:**

Meaning in life is one of the psychological domains that is most severely affected in patients with life-threatening illnesses. The importance of meaning-making mandates the development of reliable tools to assess this construct. Steger's Meaning in Life Questionnaire (MLQ) is one of the most valid and reliable instruments that determines the search for and presence of meaning in life. The present study was conducted to provide psychometric data on the MLQ in a sample of patients with life-threatening illnesses.

**Methods:**

The MLQ was completed by 301 patients (aged 20–80 years) diagnosed with life-threatening illnesses (cancer and multiple sclerosis) and referred to hospitals. Confirmatory factor analysis and Pearson's correlation test were used to determine the construct validity of the questionnaire.

**Results:**

The confirmatory factor analysis supported the original two-factor model of the MLQ, comprised of the presence of meaning (five items) and search for meaning (five items). The responses to the MLQ did not differ by sociodemographic factors. Most importantly, contrary to previous findings, the correlation between the two subscales, i.e., search for meaning and presence of meaning, was significant and positive.

**Conclusion:**

The results showed that the MLQ is a valid and reliable measure for assessing meaning in life that can be applied in research on meaning in life among other patient populations.

## 1. Introduction

Steger et al. [1] defined meaning in life as the extent to which people comprehend, see the significance in, and make sense of their lives as well as the degree to which they perceive themselves to have a purpose or mission in life [2].

In recent years, research on the construct of meaning has been a focus of mental health studies, and the consequences of meaning in life in different areas such as psychological functioning [3]; coping, adjustment, stress reduction [4, 5]; and spiritual wellbeing [6] are well supported in empirical research. Meaning in life is a vital psychological element of resilience against traumas and coping with disasters that provides individuals with the sense that their lives matter [7–12] even in times of hardship. Recent studies have well demonstrated that meaning-making can lead individuals to successful coping with their changing life conditions [13].

According to the literature, confronting stressful life events, accompanied by various losses, challenges the individual's desire to perceive the world as meaningful and predictable and might thus trigger a search for meaning [9, 14, 15]. It is the meaning attributed to such events that ascertain their significance and how the individual responds [16, 17].

Meaning in life is one of the psychological domains that is most severely affected in patients with a life-threatening illness [18–20]. After their diagnosis, these patients confront several forms of loss, with the most significant one being the loss of the sense of meaningfulness of the world [21]. The loss of previous stability in life can stimulate a psychological quest for making sense of the new existence marked by uncertainty and the possibility of premature death [22]. Making meaning thus becomes central to adaptation to life-threatening illnesses [10, 11], and searching for a positive meaning in a threatening situation can promote the adaptation process, therefore resulting in resilient responses [23].

The importance of meaning-making mandates the development of reliable tools to assess this construct. An increasing number of meaning in life instruments have been published every decade that assess the extent of experienced meaning in life, the lack thereof, sources of meaning, search for, making of, commitment to, and structure of meaning [24].

The popular measures within the context of illness that have good psychometric properties include the Meaning in Life Questionnaire (MLQ) [1], the Sense of Coherence Scale [25], the Spiritual Meaning Scale [26], the Purpose in Life Test [27], and the Life Attitude Profile [28].

The striking features of the MLQ, however, are assessing the main components of meaning separately (search for and presence of meaning), a stable factor structure [1] and, most importantly, being brief and facilitating quick response for the patients in the present study.

In a systematic review, Brandstätter et al. offer a compilation of the existing instruments on meaning in life and report MLQ as one of the quantitative measures with good psychometric properties, such as appropriateness, construct validity, item generation, concept definition, and reliability [24].

The MLQ consists of ten items within two subscales, namely, the Presence of Meaning and the Search for Meaning subscales. The respondents rate their degree of agreement with the items on a 7-point scale, ranging from 1 (“absolutely untrue”) to 7 (“absolutely true”). This questionnaire has no item overlap with other relevant measures of meaning in life and differentiates between the degree to which the individual reports meaning in life and the degree to which they are seeking it. The assessment of the validity of the MLQ showed adequate convergent, discriminate, and structural validity and acceptable fit and reliability indices. The MLQ correlates with some variables, such as personality and well-being, and the two factors have adequate reliability and internal consistency [1].

Over a decade ago, the MLQ became the most widely used measure of meaning in life in a variety of populations and diverse cultures. This questionnaire has been investigated in various healthy populations as well, including college students [29, 30] and healthy adults [31, 32]. The review of literature showed that, concerning negative stressful life events, the MLQ has been examined in displaced populations [33], people diagnosed with serious mental illnesses [34], the caregivers of patients with chronic illnesses [35], and students from earthquake-stricken areas [36].

It seems that the population of patients with life-threatening illnesses is overlooked in the MLQ literature, and no evidence was found on the empirical evaluation of the MLQ among patients with life-threatening illnesses. It should be noted, however, that apart from the MLQ, studies on meaning in life for populations with an experience of trauma or in hardship are generally scarce [33] and this subject therefore needs further consideration.

Given the importance of the meaning in life construct to people experiencing life threatening illnesses, the present study was conducted to provide psychometric data on the MLQ in a sample of patients and could help clarify the existing data on the psychometric properties of this tool and gather further facts about its construct validity.

## 2. Methods

### 2.1. Participants

The study included 301 patients with life-threatening illnesses undergoing treatment for cancer and multiple sclerosis. As for participants' demographic details, 201 were cancer patients, and 100 were MS patients; 218 were female, and 83 were male, the age range was 20–80 years.  Table 1 presents the sociodemographic profile of the samples. Data were collected from the patients who had undergone treatment at the Oncology Center of Imam Hossein Hospital and Rofeide Rehabilitation Clinic in Tehran.

All the patients and their caregivers were informed about the specific purpose of the study and provided informed written consent. The patients who were aware of their accurate diagnosis and stage of disease were asked to complete the questionnaire if they consented to participate in the study. They each completed the questionnaire in a self-reported manner. There was no time limit for completing the questionnaire, and the process took about 10 to 15 minutes. The inclusion criteria were as follows: (1) age above 18 years; (2) being aware of their diagnosis; and (3) being interested in participation in the study. This study was approved by the Medical Ethics Committee of the University of Social Welfare and Rehabilitation Sciences, Tehran, Iran.

### 2.2. Instruments

The sociodemographic questionnaire: this form collected the demographic details of each participant, including gender, age group, type of cancer, stage of disease, and education. The Meaning in Life Questionnaire (MLQ): the MLQ is a ten-item instrument consisting of two different constructs. They are as follows: the Presence of Meaning (MLQ-P; e.g., “My life has a clear sense of purpose”) and the Search for Meaning (MLQ-S; e.g., “I am seeking a purpose or mission for my life”). Each dimension of this instrument is measured by five items rated on a 7-point Likert scale. The scores range from 5 to 35, and higher scores represent higher levels of presence of meaning and search for meaning. One item in the purpose of meaning subscale (item 9) is reverse coded. In the normative sample, both subscales had Cronbach's alpha values between 0.82 and 0.88 and a one-month test-retest stability of 0.70 (MLQ-P) and 0.73 (MLQ-S) [1].

### 2.3. Data Analysis

The construct validity of the MLQ was examined using the confirmatory factor analysis (CFA). CFA was performed in LISREL 8.8 (Scientific Software International, Skokie, IL). Several indices were used to evaluate the model's goodness of fit: the chi-square test of significance (*χ*^2^), the root-mean-square error of approximation (RMSEA), the Normed Fit Index (NFI), the Nonnormed Fit Index (NNFI), the Goodness-of-Fit Index (GFI), the Adjusted Goodness-of-Fit Index (AGFI), the Standardized Root Mean Square Residual (SRMR), and the Comparative Fit Index (CFI). According to the guidelines, CFI values greater than 0.90 have been recommended as indicative of a good fit of the model [36, 37], and RMSEA values must be 0.08 or less. GFI values greater than 0.95, AGFI values greater than 0.90, SRMR values less than 0.08, and (N)NFI values greater than 0.95 are all deemed acceptable and demonstrate the good fit of the model [38, 39].

The reliability, homogeneity, and internal consistency were also assessed using the interitem correlation and Cronbach's alpha value. The MANOVA was performed to examine the effect of the demographic variables on the MLQ. The statistical analyses were performed using IBM SPSS-version 21.

## 3. Results

### 3.1. Confirmatory Factor Analysis (CFA)

Since the two-factor structure of the MLQ was already established based on several types of research, studies, and theoretical bases, CFA was used to test the validity of the two-factor structure of the scale. To examine the suitability of the two-factor structure of the MLQ, both absolute and relative fit indices were measured.  Table 2 presents the goodness-of-fit values of the model. Overall, the two-factor model of the MLQ was confirmed using the present dataset. RMSEA was below the maximum acceptable value of 0.08. The goodness of fit using RMSEA shows a marginally acceptable fit for the model. The GFI of 0.99 also indicates an acceptable fit. The standardized regression weights for each item were also above 0.7, except for item 9 [40], which seems to be problematic, as previous studies have also suggested its removal from the MLQ [31, 41, 42].

**Figure 1:**
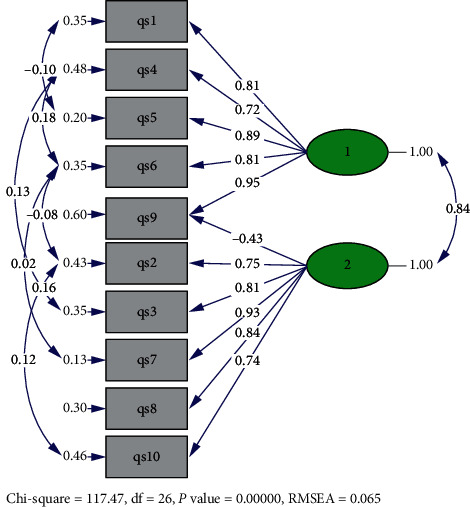
MLQ confirmatory factor analysis four-factor model. The correlation between the search and presence subscale is significant at the *P* < 0.01 level and the correlation between items are significant at the *P* < 0.01 and *P* < 0.05 levels. MLQ = Meaning in Life Questionnaire.

Figure 1 presents the path diagram of the model. The loading of the items on the respective factors ranged from 0.7 (item 4) to 0.9 (item 9), and the conclusion was that the data fit the preferred, original, two-factor model reasonably well. There was also a positive association between the presence and search subscales (*r* = 0.61, *P* < 0.001). These findings will be further explained in Section 4 of this article.

### 3.2. Internal Consistency

To assess the internal consistency of the MLQ, Cronbach's alpha of the scale and subscales were computed. The alpha coefficient for the MLQ was 0.90, and its subscales also revealed high levels of internal consistency (0.84 for the MLQ-P and 0.88 for the MLQ-S; Table 3). In general, an internal consistency of *α* ≥ 0.90 is considered excellent [43].

All the MLQ-P subscale items correlated significantly and positively with each other, with values ranging from 0.40 (between items 5 and 9) to 0.68 (between items 6 and 4). The same pattern was observed for all the MLQ-S subscale items, with values ranging from 0.50 (between items 3 and 10) to 0.72 (between items 8 and 7). Furthermore, all the subscale items correlated positively with each other, with values ranging from 0.13 (between items 9 and 10) to 0.72 (between items 7 and 8). In addition, the correlation of item 9 with the sum of the scores of the MLQ-S items was 0.28. As noted in the CFA section, it seems that item 9 is somehow problematic and is not perfectly homogeneous with the MLQ-S subscale items.  Table 4 presents Pearson's correlations among the MLQ items.

### 3.3. MLQ and the Correlation between Its Demographic Variables

The relationship of the MLQ-P and MLQ-S was examined with the demographic variables. The scores obtained for the MLQ-S and MLQ-P did not differ by disease type (Wilks' lambda = 0.99, *F* = 1.4, *P*=0.24), gender (Wilks' lambda = 0.99, *F* = 0.92, *P*=0.39), education (Wilks' lambda = 0.97, *F* = 0.57, *P*=0.85), marital status (Wilks' lambda = 0.99, *F* = 0.33, *P*=0.91), age (Wilks' lambda = 0.98, *F* = 0.36, *P*=0.96), disease duration (Wilks' lambda = 0.97, *F* = 0.74, *P*=0.68), cancer stage (Wilks' lambda = 0.98, *F* = 0.61, *P*=0.72), and MS stage (Wilks' lambda = 0.95, *F* = 0.74, *P*=0.61). For a better comprehension of the results, Table 5 reports the descriptive statistics of the MLQ and its subscales based on the demographic variables.

## 4. Discussion

This study was conducted to determine the psychometric properties of the Meaning in Life Questionnaire (MLQ) among patients with life-threatening illnesses. The factor structure, internal consistency, and internal validity of the MLQ were examined. As for the question of whether or not the MLQ has construct validity, a confirmatory factor analysis was conducted to explore its factor structure and construct validity. CFA supported the two-factor model, including the Presence of Meaning and Search for Meaning subscales, consistent with the results of several previous studies [1, 29–34, 40]. The results also showed that item 9 (“My life has no clear purpose”), which is the only reversed item in the MLQ-P, had a significantly lower factor loading (*P* < 0.05) onto the other items on this subscale (MLQ-P = 0.18, MLQ-S = 0.44) and seems to be a problematic item. This item also demonstrated a low correlation with the MLQ-S items, especially with items 2, 8, and 10 (*r* = 0.25, 0.19, and 0.13). In line with the present study, some scholars emphasized the inappropriate assignment of item 9 to the MLQ-P subscale [33, 42, 43]. Schutte et al. discussed the poor performance of item 9 based on the Rasch rating scale model and suggested the removal of this item [31].

In their study on older adults, Hallford et al. also reported that this item has a poor loading and discriminate validity, and after its removal, the model fit improved significantly [42]. A significant aspect of the present study is that, despite the poor performance of item 9, the final model had a significantly good fit (RMSEA = 0.06). To maintain the scale's internal consistency [1], however, this item was retained in the questionnaire in this study. The poor performance of item 9 can be explained by noting its negative nature and the differences in its scoring system in the MLQ-P subscale, which makes the comprehensibility of its content relatively difficult for the participants compared to the more straightforward formulation of the other MLQ items [33, 41].

Somewhat unexpectedly, contrary to previous findings, which have mostly shown a reverse or negative correlation [1, 29, 32, 41, 44, 45] or no significant relationships [34] between the MLQ subscales, the presence and search for meaning subscales in the present study correlated significantly with each other (*r* = 0.61). The participants also reported a high average score in both factors (*P*=27.1, *S* = 28.6).

The data obtained in this study differed significantly from the majority of the studies, and few similar findings were reported in some other populations, such as displaced persons, clinical samples, and older adults, in which a positive correlation was found between the presence and search subscales [33, 46, 47].

The present findings can be explained within five dimensions. First of all, life-threatening illnesses, such as cancer, and MS comprise an uncertain situation that is beyond the individual's control [21, 22, 48], and patients may experience an existential struggle in the face of the unpredictability of life in such situations. As a result of the uncertainty, vulnerability, and progressiveness of the situation, even if patients have actually achieved meaning in their life, explaining their challenging conditions may be insufficient and they thus remain in a state of continuous search for meaning.

Second, the meaning-making process is, by nature, an ongoing and unstable process [40, 49] that is strongly context dependent [46] and does not stop even after the achievement of meaning because just then, the patients further uncover the crucial role of this process in adjusting to illness and may thus continue to express their desire for its search [46].

Third, even if the patients achieve a satisfactory and desirable meaning in their life and lead a meaningful life, they may still be looking for a deeper and greater meaning [40, 50, 51] because the human tendency toward searching for deeper and greater meaning is undeniable and people are motivated to not only find a meaning but to also constantly search for new ones [32].

Fourth, people seem to discover and experience meaning in various domains of life [13]. After diagnosis with a severe illness, patients often lose only some aspects of the meaning they have formerly ascribed to life and then initiate their search for meaning. Consequently, during the fight against illness, patients may preserve some domains of the meaning they have been experiencing and living with before their diagnosis. An important matter is that the MLQ cannot assess these dimensions and the deeper facets of the construct.

Fifth, when considering the search for meaning as a coping and adaptation strategy, being involved in this search in spite of having already found some meaning can help sustain the patient's psychological functioning during his/her struggle with illness. Frankl argued that meaning in life is an intrapersonal resource that can be used to maintain well-being and adaptive functioning [52]. Moreover, many scholars have shown that experiencing meaning is more important for psychological adjustment during illness and can act as a buffer for stress [49, 53].

To summarize, it seems that search for meaning does not indicate the absence of meaning or vice versa. Since the concept of meaning-making is broad and complicated, examining the construct of meaning without considering this concept and the components of meaning is very misleading.

In this study, no significant differences were found in the search for meaning or presence of meaning based on sociodemographic variables such as age, education, gender, disease duration, and marital status. This finding is consistent with the results of some studies [1, 33] and contradicts some others that have reported a significant correlation between the MLQ and sociodemographic variables [23].

This study had a number of limitations that should be taken into account when interpreting its findings. The main limitation might be the selection bias in the sampling process, which is the result of selecting only volunteering patients. The study population may therefore not be an exact representative of the target population since, compared to those unwilling to respond to the questionnaire or participate in the study, volunteering patients may have different characteristics, such as being open and welcoming. Due to these potential characteristics in the participants, the research findings may be confounded by the selection bias and their internal validity is therefore uncertain. Also, the convenience and nonrandom method of sampling used might have made the generalization and external validity of the data problematic [54].

Regarding the validation procedure, this study did not examine any other measures to complement the results on the MLQ's discriminant and concurrent validity and did not include a test-retest reliability check either. The researchers therefore propose that the MLQ be evaluated in future studies alongside other psychological variables such as mental health, coping styles, and resilience.

To better understand the changes over time in the meaning-making process, a longitudinal design may help clarify the dynamic relationships between the search for and presence of meaning. The generalization of the present findings is contingent on further research in the future, and larger populations as well as other patient groups with different threatening illnesses are recommended to be studied in order to verify the validity of the MLQ. Although searching for meaning leads to a greater meaning [32], the present study was unable to determine whether or not the patients' process of search for and achievement of meaning were healthy and functional.

The following questions can guide future studies on the MLQ and, more generally, the concept of meaning in life:

  What variables affect the dynamic relationships between searching for meaning and the presence of meaning?
  How can we understand whether or not our meaning-making process is healthy and functional? What variables mediate between threatening situations and the need to make sense of life and search for meaning?

## 5. Conclusion

Highly stressful life events tend to disturb people's sense of meaning in life [5]. The present study was conducted as a preliminary examination of the reliability and validity of the Meaning in Life Questionnaire among patients with life-threatening illnesses. Further research is required to sufficiently establish the reliability and validity of this questionnaire for broader samples of patients.

## Figures and Tables

**Table 1:** Demographic characteristics of the samples (*N* = 301).

Variables	Frequency	Percentage
Illness		
Cancer	201	66/8
MS	100	33/2

Gender		
Female	218	72/4
Male	83	27/6

Age		
20–30	39	13/0
30–40	92	30/6
40–50	83	27/6
50–60	51	16/9
60–70	36	12/0

Disease duration		
0–5	218	72/4
5–10	41	13/6
10–15	21	7/0
15–20	13	4/3
20–25	8	2/7

MS stage		
1 disable organ	37	37/0
2 disable organs	26	26/0
3 disable organs	11	11/0
4 disable organs	3	3/0
No disable organs	23	23/0

Cancer stage		
Stage 1	64	31/8
Stage 2	52	25/9
Stage 3	27	13/4
Stage 4	58	28/9

Marriage		
Single	74	24/6
Married	205	68/1
Divorced	10	3/3
Widowed	12	4/0

Education		
School	83	27.6
Diploma	110	36.5
University	108	35.9

**Table 2:** Fit indices related to CFA of the MLQ.

Model	Goodness-of-fit statistics							
	*χ* ^2^ (df), *P*	GFI	AGFI	NFI	NNFI	CFI	RMSEA (90% CI)	SRMR
Two factors	117.47 (26), *P* < 0.001	0.93	0.85	0.99	0.99	0.99	0.065 (0.04, 0.08)	0.05

CFA: confirmatory factor analysis; MLQ: meaning in life questionnaire; *X*^2^: normal theory, weighted least squares chi-square; GFI: Goodness-of-Fit Index; AGFI: Adjusted Goodness-of-Fit Index; NFI: Normed Fit Index; NNFI: Nonnormed Fit Index; CFI: Comparative Fit Index; RMSEA: root-mean-square error of approximation; CI: confidence interval; SRMR: standard root-mean-square residual.

**Table 3:** Internal consistency coefficients of the MLQ subscale.

Factor	Item number	Cronbach's alpha
1	5	0.84^*∗*^
2	5	0.88^*∗*^
Total	10	0.90^*∗*^

MLQ: Meaning in Life Questionnaire. ^*∗*^*p* < 0.05 (2-tailed).

**Table 4:** Mean score, standard deviation, item total, and interitem correlations of the MLQ.

Items	M (SD)	Item total	1	2	3	4	5	6	7	8		
1	5.2 (1.5)	0.67	__									
2	5.7 (1.5)	0.67	0.513^*∗∗*^	__								
3	5.7 (1.5)	0.73	0.536^*∗∗*^	0.703^*∗∗*^	__							
4	5.4 (1.6)	0.62	0.567^*∗∗*^	0.389^*∗∗*^	0.477^*∗∗*^	__						
5	6.0 (1.5)	0.74	0.529^*∗∗*^	0.563^*∗∗*^	0.583^*∗∗*^	0.499^*∗∗*^	__					
6	5.3 (1.7)	0.68	0.619^*∗∗*^	0.367^*∗∗*^	0.442^*∗∗*^	0.687^*∗∗*^	0.587^*∗∗*^	__				
7	5.9 (1.4)	0.77	0.536^*∗∗*^	0.611^*∗∗*^	0.690^*∗∗*^	0.405^*∗∗*^	0.698^*∗∗*^	0.538^*∗∗*^	__			
8	5.7 (1.6)	0.64	0.398^*∗∗*^	0.531^*∗∗*^	0.585^*∗∗*^	0.317^*∗∗*^	0.568^*∗∗*^	0.433^*∗∗*^	0.723^*∗∗*^	__		
9	5.1 (2.0)	0.44	0.417^*∗∗*^	0.255^*∗∗*^	0.335^*∗∗*^	0.505^*∗∗*^	0.404^*∗∗*^	0.487^*∗∗*^	0.294^*∗∗*^	0.193^*∗∗*^	__	
10	5.5 (1.7)	0.55	0.314^*∗∗*^	0.585^*∗∗*^	0.509^*∗∗*^	0.272^*∗∗*^	0.476^*∗∗*^	0.344^*∗∗*^	0.589^*∗∗*^	0.581^*∗∗*^	0.137^*∗*^	^___^

^*∗∗*^ *P* < 0.01; ^*∗*^*P* < 0.05.

**Table 5:** Descriptive statistics for MLQ and subscales regarding demographic variables.

Variables	MLQ	MLQ-P	MLQ-S
	Mean (SD)	Mean (SD)	Mean (SD)
Diseases			
Cancer	56.42 (11.74)	27.56 (6.69)	28.86 (6.44)
MS	54.35 (12.39)	26.17 (6.85)	28.18 (6.83)
All	55.73 (11.97)	27.10 (6.71)	28.63 (6.52)

Age			
20–30	56.08 (8.89)	27.44 (5.70)	28.64 (5.39)
30–40	54.89 (13.53)	26.47 (7.57)	28.42 (7.15)
40–50	56.06 (11.37)	27.05 (6.39)	29.01 (6.66)
50–60	56.69 (11.29)	27.76 (6.20)	28.92 (6.39)
60–70	54.07 (13.28)	27.11 (7.19)	27.61 (6.54)

Gender			
Male	56.80 (12.49)	27.90 (6.76)	28.89 (6.69)
Female	55.33 (11.78)	26.79 (6/75)	28.54 (6.53)

Marital status			
Single	53.75 (2.49)	26.12 (1.40)	27.62 (1.37)
Married	55.25 (3.07)	27.37 (1.73)	28.15 (1.69)
Divorced	56.85 (4.15)	27.07 (2.34)	29.78 (2.29)
Widowed	57.63 (3.96)	27.77 (2.23)	29.86 (2.18)

Education			
School	55.12 (11.97)	26.70 (6.92)	28.42 (6.52)
Diploma	55.68 (13.19)	27.13 (7.26)	28.55 (7.11)
University	56.73 (10.69)	27.37 (6.14)	28.88 (6.06)

MS stage			
1 disable organ	54.89 (12.73)	26.50 (6.19)	28.39 (7.63)
2 disable organ	53.42 (13.22)	25.35 (7.69)	28.08 (6.86)
3 disable organ	52.71 (9.20)	24.43 (6.14)	28.29 (5.04)
No disable organ	55.50 (13.09)	27.58 (7.30)	27.92 (6.81)

Cancer stage			
Stage 1	57.19 (9.70)	28.14 (5.99)	29.05 (5.40)
Stage 2	55.23 (15.31)	27.00 (7.42)	28.23 (8.54)
Stage 3	59.22 (10.29)	29.07 (6.59)	30.15 (5.43)
Stage 4	55.33 (10.60)	26.71 (6.76)	28.62 (5.77)

Disease duration			
0–5	56.06 (11.78)	27.33 (6.76)	28.73 (6.76)
5–10	56.05 (11.76)	27.00 (6.38)	27.00 (6.38)
10–15	53.24 (12.16)	25.57 (7.48)	25.57 (7.48)
15–20	51.92 (15.21)	24.54 (7.93)	24.54 (7.93)
20–25	58.09 (6.08)	29.73 (4.04)	29.73 (5.06)

## Data Availability

The data used to support the findings of this study are available from the corresponding author upon request.
